# Comparison of the predictive ability of albuminuria and dipstick proteinuria for mortality in the Japanese population: the Yamagata (Takahata) study

**DOI:** 10.1007/s10157-015-1193-0

**Published:** 2015-11-05

**Authors:** Hiroko Sato, Tsuneo Konta, Kazunobu Ichikawa, Natsuko Suzuki, Asami Kabasawa, Kazuko Suzuki, Atsushi Hirayama, Yoko Shibata, Tetsu Watanabe, Takeo Kato, Yoshiyuki Ueno, Takamasa Kayama, Isao Kubota

**Affiliations:** Department of Cardiology, Pulmonology, and Nephrology, Yamagata University School of Medicine, 2-2-2, Iida-Nishi, Yamagata, 990-9585 Japan; Global COE, Yamagata University School of Medicine, Yamagata, Japan

**Keywords:** Albuminuria, Proteinuria, Mortality, Population, Cohort

## Abstract

**Background:**

Albuminuria and proteinuria are known risk factors for premature death. This study compared the ability of albuminuria and proteinuria to predict mortality in a community-based population.

**Methods:**

We evaluated the urinary albumin creatinine ratio (ACR) and proteinuria by dipstick at a baseline survey and examined the association between the 7-year mortality and three categories (albuminuria [ACR ≥ 30 mg/g], trace proteinuria, and ≥[1+] proteinuria) in 3446 Japanese subjects at a local health check.

**Results:**

Albuminuria, ≥trace proteinuria, and ≥(1+) proteinuria were identified in 514 (14.9 %), 290 (8.4 %), and 151 (4.4 %) subjects, respectively. There were 138 deaths during the follow-up period, including 41 cardiovascular deaths. A Kaplan–Meier analysis showed that all-cause mortality significantly increased along with the increase in ACR and proteinuria levels (log-rank *P* < 0.01). The mortality rate (deaths per 1000 person-year) was higher in subjects with albuminuria (12.8), ≥trace proteinuria (12.6), and ≥(1+) proteinuria (16.2) than in all subjects (6.9). A Cox proportional hazard model analysis showed that all three categories were significant predictors of all-cause mortality in the unadjusted model, although after adjustment for possible confounders, a significant association was observed only with albuminuria. Albuminuria, but not proteinuria, was a significant predictor of cardiovascular mortality in both the unadjusted and adjusted models.

**Conclusion:**

Albuminuria had a high prevalence and was strongly associated with mortality, as compared with proteinuria by dipstick, suggesting that albuminuria might be a superior predictor of poor prognosis in the Japanese population.

## Introduction

The number of patients with chronic kidney disease (CKD) is growing worldwide, and CKD is a significant risk factor for not only end-stage kidney disease (ESKD) [1], but also cardiovascular disease (CVD) and premature death [2, 3]. Therefore, the detection of CKD at the earliest opportunity is required to prevent a poor outcome. The CKD is characterized by two major components: (1) urinary abnormalities such as albuminuria or proteinuria, and (2) glomerular filtration rate (GFR) <60 mL/min/1.73 m^2^ [4].

To detect albuminuria/proteinuria in spot urine samples, two methods are mainly utilized, namely, the direct measurement of urinary albumin concentration and the semiquantitative evaluation of proteinuria by dipstick. However, our previous study reported that trace proteinuria detected by dipstick might be used as a useful indicator for albuminuria [5].

A recent analysis revealed that high-grade albuminuria and proteinuria are associated with an increased risk of all-cause and cardiovascular mortality, independent of renal function [2, 3, 6]. Accordingly, to screen for high-risk individuals, either albuminuria or proteinuria is evaluated during the health check.

Until now, no study has directly compared the ability of these methods to predict mortality. To address this issue, we conducted the longitudinal observational study in a Japanese community.

## Subjects and methods

The Yamagata (Takahata) study was a part of the ongoing Molecular Epidemiological Study, utilizing the resources of the Regional Characteristics of 21st Century Centers of Excellence (COE) program and the Global COE in Japan. The study enrolled subjects at a community-based annual health check, and all inhabitants of Takahata, a town in northern Japan (total population 26,026), who were ≥40 years of age were invited to participate. From June 2004 through November 2006, 3520 subjects took part in the program and agreed to participate in the study. We followed them for 7 years and examined the association between albuminuria and proteinuria [≥trace proteinuria, ≥(1+) proteinuria] and all-cause and cardiovascular mortality. The institutional ethics committees of the Yamagata University School of Medicine and the town of Takahata approved this study (24 May 2004, No. 3), and all subjects provided written informed consent. The procedures were performed in accordance with the Helsinki Declaration. Details regarding the study design, recruitment procedure, and population profile have been published elsewhere [7]. Seventy-four subjects were excluded from the analysis because of incomplete urinary data or withdrawal of agreement. Data from a total of 3446 subjects were entered into the final statistical analysis. There were 1552 (45 %) men and 1894 (55 %) women, and the mean age was 63 years.

To investigate the association between albuminuria, proteinuria and prognosis, a follow-up survey was performed annually until the end of 2010 [6]. The causes of death were determined by reviewing death certificates through the end of 2010. The death code (International Classification of Diseases, 10th Revision) and the date and place of death were reviewed.

### Measurements

At baseline, the survey subjects used a self-reported questionnaire to document their medical history, current use of medications, and clinical symptoms. Systolic and diastolic blood pressure was determined using a mercury manometer with subjects in a sitting position, after resting for at least 5 min. Hypertension was defined as a systolic blood pressure ≥140 mmHg, a diastolic blood pressure ≥90 mmHg, or the use of antihypertensive medication. Subjects with a body mass index of ≥25.0 kg/m^2^ were categorized as obese [8]. The presence of diabetes was defined as a plasma glucose level of ≥126 mg/dL, HbA1c 6.5% (Japanese Diabetes Society value), or the use of antidiabetic medication. Hypercholesterolemia was defined as a serum total cholesterol ≥220 mg/dL and/or the use of antihyperlipidemic medication.

The measurement of urinary albumin creatinine ratio (ACR) and urinalysis by dipstick (Ames Multistix, Bayer Diagnostic, Victoria, Australia) were performed using a single-spot urine specimen collected early in the morning after overnight fasting. Urinary albumin concentrations were determined by immunoturbidimetry. Albuminuria was defined as an ACR ≥ 30 mg/gCr [4, 6]. The results of the urine test by dipstick were interpreted by a physician or a physician’s assistant and were recorded as (−), trace, (1+), (2+), or (3+). Proteinuria are defined as ≥(1+) proteinuria by dipstick. Serum creatinine level was measured by an enzymatic method. GFR was estimated using an equation for Japanese subjects [9]. 24 h urinary sodium excretion was estimated by Kawasaki’s equation using a spot urine specimen [10].

### Statistical analysis

Data are expressed as the mean ± SD unless otherwise indicated. A Kaplan–Meier method was used to calculate the cumulative probability. A Kaplan–Meier analysis with log-rank test and both unadjusted and adjusted Cox-proportional hazard model analyses were performed to examine the relationship between albuminuria, proteinuria, and all-cause and cardiovascular death. To verify the proportional hazard assumption of albuminuria and proteinuria on mortality, we created the graph of the log [−log (survival)] vs. log of survival time and confirmed that the graph of these parameters resulted in parallel curves. For continuous variables including age, eGFR and 24 h urinary sodium excretion, we converted them to categorical variables (i.e., per 10 year increase for age, per 15 mL/min/1.73 m^2^ increase for eGFR, and per 1SD increase [54 mEq/day] for 24 h urinary sodium excretion, respectively) and verified the proportional hazard assumption. The sensitivity, specificity, and area under a receiver operating characteristic (ROC) curve (AUC) for all-cause and cardiovascular mortality was evaluated. In the analysis to calculate sensitivity, specificity and AUC, the subjects censored during 7-year follow-up period by moving out [*n* = 37 (1.1 %)] was included in those without events. In this ROC analysis, nominal variables (albuminuria and proteinuria) was converted to numerical values (1 or 0): the positive group of urinary parameters (i.e., albuminuria (+), ≥trace proteinuria, and ≥(1+) proteinuria) as 1 and the corresponding reference group (i.e., albuminuria (−), proteinuria (−), and ≤trace proteinuria) as 0, respectively and outcome (mortality) was converted to binary variables (1 or 0): all-cause or cardiovascular death (+) as 1 and all-cause or cardiovascular death (−) as 0, respectively. In the adjusted model, the hazard ratio (HR) was adjusted for age, gender, alcohol consumption, smoking, obesity, hypertension, diabetes, hypercholesterolemia, estimated GFR, and urinary sodium excretion. Furthermore, we performed the subgroup analysis stratified by the covariates including age (≥65 years), gender, hypertension, diabetes, obesity, hypercholesterolemia, smoking, and alcohol consumption, eGFR (<60 ml/min/1.73 m^2^), and 24 h urinary sodium excretion (≥200 mEq/day). A significant difference was defined as *P* < 0.05. All statistical analyses were performed using JMP version 10 (SAS Institute Inc., Cary, NC, USA).

## Results

The prevalence of albuminuria, ≥trace proteinuria, and ≥(1+) proteinuria was 14.9, 8.4, and 4.4 %, respectively, and clinical characteristics are presented in Table 1. The subjects with albuminuria or proteinuria were older and showed a higher prevalence of comorbidities such as hypertension, obesity, smoking, and diabetes compared with the total population.

**Table 1:** Comparison of the baseline characteristics of the study subjects

	All subjects	Albuminuria (+)	Albuminuria (−)	≥Trace proteinuria	Proteinuria (−)	≥(1+) proteinuria	≤Trace proteinuria
	(*n* = 3446)	(*n* = 514)	(*n* = 2932)	(*n* = 290)	(*n* = 3156)	(*n* = 151)	(*n* = 3295)
Male (%)	45.0	48.1	44.6	56.2*	44.0	63.3*	44.3
Age (years)	62.6 ± 10.4	66.8 ± 9.8*	61.8 ± 10.3	63.5 ± 11.0	62.4 ± 10.3	63.9 ± 11.1	62.5 ± 10.3
Systolic BP (mmHg)	134.4 ± 15.8	142.6 ± 15.4*	132.9 ± 15.4	139.7 ± 16.0*	133.8 ± 15.7	140.4 ± 14.6*	134.1 ± 15.8
Diastolic BP (mmHg)	79.6 ± 10.1	82.2 ± 11.0*	79.1 ± 9.9	82.0 ± 11.8*	79.4 ± 9.9	82.6 ± 10.6*	79.5 ± 10.1
Serum creatinine (mg/dL)	0.68 ± 0.22	0.73 ± 0.44*	0.65 ± 0.15	0.78 ± 0.55*	0.67 ± 0.16	0.86 ± 0.73*	0.67 ± 0.16
eGFR (mL/min/1.73 m^2^)	81.5 ± 16.5	79.1 ± 21.3*	82.0 ± 15.5	77.3 ± 20.9*	81.9 ± 16.0	75.3 ± 21.9*	81.8 ± 16.2
Hemoglobin (g/dL)	13.7 ± 1.5	13.9 ± 1.5*	13.7 ± 1.5	14.0 ± 1.6*	13.7 ± 1.5	14.2 ± 1.5*	13.7 ± 1.5
Uric acid (mg/dL)	5.1 ± 1.4	5.4 ± 1.5*	5.0 ± 1.3	5.5 ± 1.4*	5.0 ± 1.3	5.6 ± 1.5*	5.0 ± 1.3
e24UNa (mEq/day)	201 ± 54	209 ± 63*	199 ± 52	169 ± 51*	203 ± 54	171 ± 52*	202 ± 54
Hypertension (%)	44.4	65.1*	40.8	62.3*	42.7	64.2*	43.4
Obesity (%)	30.3	41.4*	28.3	40.7*	29.3	43.0*	29.7
Smoker (%)	32.4	35.8	31.8	40.7*	31.7	49.7*	31.6
Alcohol consumption (%)	41.6	41.2	41.7	47.2*	41.1	49.0	41.3
Diabetes (%)	5.4	15.2*	3.7	15.3*	4.5	18.1*	4.8
Hypercholesterolemia (%)	33.5	36.4	33.0	35.5	33.4	33.1	33.6

*BP* blood pressure, *eGFR* estimated glomerular filtration rate, *e24UNa* estimated 24 h urinary sodium excretion

Values are reported as mean ± SD unless otherwise indicated. * *P* < 0.05 vs. counterparts

**Fig. 1:**
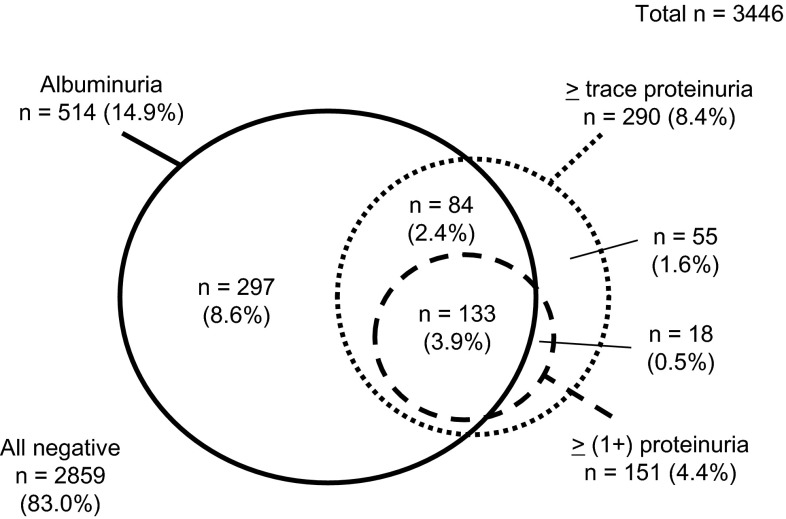
Distribution of albuminuria and proteinuria among study subjects

The distribution of subjects with albuminuria, ≥trace proteinuria, and ≥(1+) proteinuria among the studied population is shown in Fig. 1. Although the majority of subjects with ≥trace proteinuria and ≥(1+) proteinuria had albuminuria, some (25.2 % of subjects with ≥trace proteinuria and 11.9 % of subjects with ≥[1 +] proteinuria) did not. This indicates that there is a difference in the prevalence and distribution of albuminuria and proteinuria among participants at the health check.

During the follow-up period (median 6.2 years), 138 subjects (4.0 %) died. There were 41 cardiovascular deaths (1.2 %) (19 due to coronary artery disease, 10 due to stroke and 12 due to others) and 97 noncardiovascular deaths (2.8 %) (54 due to cancer, 12 due to infection, 8 due to accidents, 5 due to lung disease and 18 due to others).

**Fig. 2:**
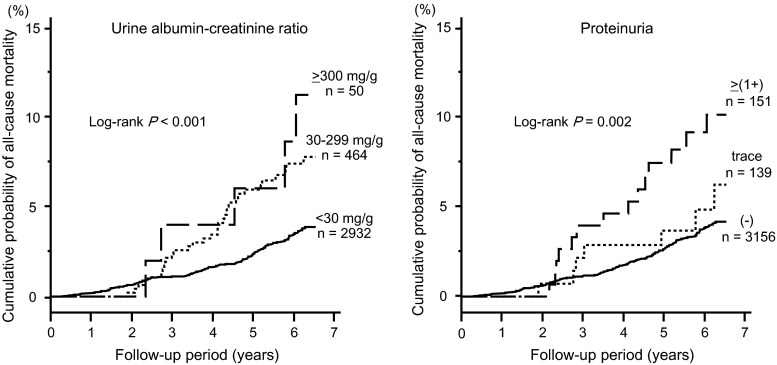
7-year all-cause mortality rate by grade of urine ACR and proteinuria

**Fig. 3:**
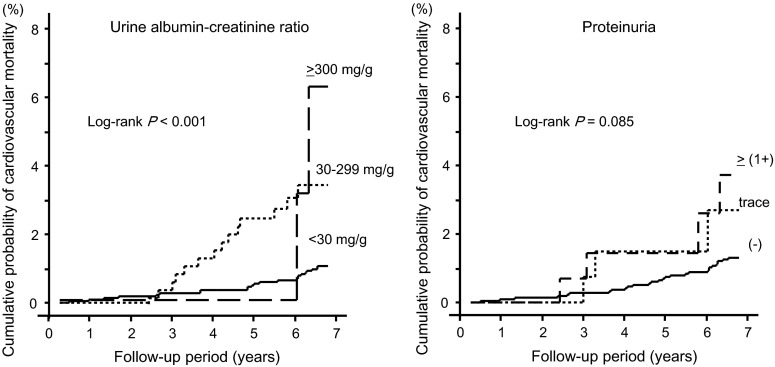
7-year cardiovascular mortality rate by grade of urine ACR and proteinuria

A Kaplan–Meier analysis showed that all-cause mortality significantly increased along with the increase in urinary albumin excretion (log-rank *P* < 0.001) and proteinuria levels (log-rank *P* = 0.002) (Fig. 2). Similarly, cardiovascular mortality increased along with the increase in urinary albumin excretion (log-rank *P* < 0.001) and proteinuria levels (log-rank *P* = 0.085) (Fig. 3). The all-cause and cardiovascular mortality rate (deaths per 1000 person-year) was 6.9 and 2.0 in all subjects, 12.8 and 5.4 in subjects with albuminuria, 12.6 and 4.2 in subjects with ≥trace proteinuria, and 16.2 and 4.6 in subjects with ≥(1+) proteinuria, respectively. The sensitivity, specificity, and AUC for all-cause mortality were 30.1, 85.9 %, 0.566 for albuminuria, 17.3 %, 92.1 % and 0.535 for ≥trace proteinuria, and 12.7, 95.9 and 0.530 for ≥1+ proteinuria, respectively. The sensitivity, specificity, and AUC for cardiovascular mortality were 39.0, 85.8, 0.624 for albuminuria, 17.1, 91.9 and 0.545 for ≥trace proteinuria, and 9.8, 95.9 and 0.528 for ≥1+ proteinuria, respectively (Table 2).

**Table 2:** The predictive performance of three cut-off values for all-cause and cardiovascular mortality

Cut-off value	All-cause mortality			Cardiovascular mortality		
	Sensitivity (%)	Specificity (%)	AUC	Sensitivity (%)	Specificity (%)	AUC
Albuminuria (+)	30.1	85.9	0.566	39.0	85.8	0.624
≥Trace proteinuria	17.3	92.1	0.535	17.1	91.9	0.545
≥(1+) proteinuria	12.7	95.9	0.530	9.8	95.9	0.528

The subjects censored during 7 year follow-up period by moving out [*n* = 37 (1.1 %)] was included in those without events

*AUC* the area under a receiver operating characteristic (ROC) curve

To examine the independent association between albuminuria/proteinuria and mortality, we performed a Cox proportional hazard analysis (Table 3). In the unadjusted model, albuminuria, ≥trace proteinuria, and ≥(1+) proteinuria were significantly associated with the 7-year all-cause mortality. However, in the multivariate model adjusted for possible confounders including age, gender, hypertension, diabetes, obesity, hypercholesterolemia, smoking, alcohol consumption, estimated GFR, and urinary sodium excretion, an independent association with all-cause mortality was observed with albuminuria (HR 1.69, 95 % confidence interval [CI] 1.11–2.54, *P* = 0.016), but not ≥ trace proteinuria (HR 1.61, 95 % CI 0.95–2.61, *P* = 0.075) or ≥(1+) proteinuria (HR 1.75, 95 % CI 0.91–3.09, *P* = 0.090). Similarly, in the multivariate model, a significant association with cardiovascular mortality was observed only with albuminuria (HR 2.49, 95 % CI 1.21–4.99, *P* = 0.014) and not with ≥ trace proteinuria (HR 1.66, 95 % CI 0.63–3.84, *P* = 0.290) or ≥(1+) proteinuria (HR 1.43, 95 % CI 0.39–4.03, *P* = 0.552).

**Table 3:** Association between albuminuria/proteinuria and all-cause and cardiovascular mortality

	Unadjusted		Adjusted^a^	
	HR (95 % CI)	*P* value	HR (95 % CI)	*P* value
All-cause mortality				
Albuminuria	2.20 (1.51–3.16)	<0.001	1.69 (1.11–2.54)	0.016
≥Trace proteinuria	2.00 (1.22–3.11)	0.007	1.61 (0.95–2.61)	0.075
≥(1+) proteinuria	2.54 (1.40–4.25)	0.003	1.75 (0.91–3.09)	0.090
Cardiovascular mortality				
Albuminuria	3.84 (2.01–7.13)	<0.001	2.49 (1.21–4.99)	0.014
≥Trace proteinuria	2.38 (0.97–5.06)	0.058	1.66 (0.63–3.84)	0.290
≥(1+) proteinuria	2.58 (0.77–6.44)	0.111	1.43 (0.39–4.03)	0.552

*HR* hazard ratio, *CI* confidence interval

^a^Adjusted for age, gender, hypertension, diabetes, obesity, hypercholesterolemia, smoking, alcohol consumption, eGFR, and estimated 24 h urinary sodium excretion

**Fig. 4:**
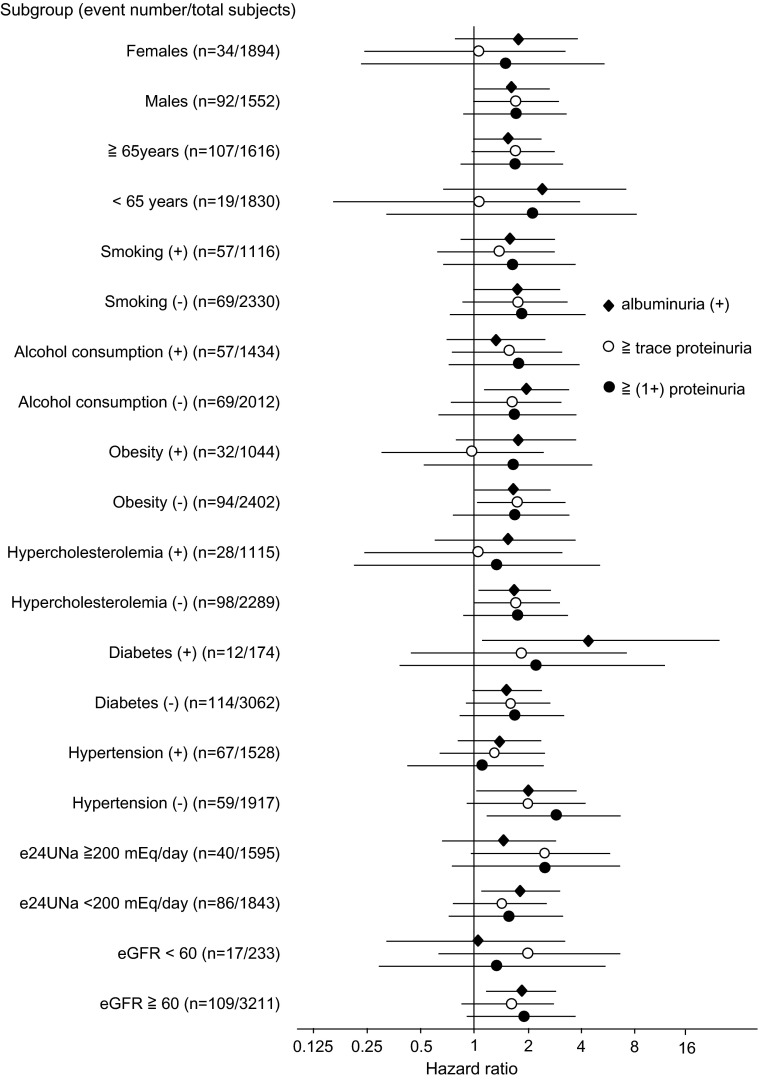
Subgroup analysis: hazard ratio of albuminuria and proteinuria for all-cause mortality The reference category is albuminuria (−) [vs. albuminuria (+)], proteinuria (−) (vs. ≥trace proteinuria), and <trace proteinuria [vs. ≥(1+) proteinuria], respectively. The *error bar* indicates 95 % confidence interval

Additionally, we performed the subgroup analysis stratified by the covariates including age (≥65 years), gender, hypertension, diabetes, obesity, hypercholesterolemia, smoking, and alcohol consumption, eGFR (<60 ml/min/1.73 m^2^), and 24 h urinary sodium excretion (≥200 mEq/day). It showed that the hazard ratio and 95 % CI for all-cause mortality of 3 categories (albuminuria, ≥trace proteinuria, and ≥(1+) proteinuria) was similar in all subgroups, except diabetic subjects that a hazard ratio was a little higher in albuminuria than that in two others (Fig. 4).

## Discussion

In this cohort study, we examined the ability of urinary albumin excretion and dipstick proteinuria to predict mortality in a local population. We found that the prevalence of albuminuria was higher than that of proteinuria by dipstick and that albuminuria, but not ≥trace proteinuria or ≥1+ proteinuria, was an independent predictor of all-cause and cardiovascular mortality.

Previous studies in the general population revealed that all-cause and cardiovascular mortality significantly increase along with the progression of albuminuria or dipstick proteinuria [2, 3, 6]. However, these studies examined the predictive ability of albuminuria or dipstick proteinuria separately, not simultaneously. To our knowledge, the direct comparison between these two indices has not been previously reported, and this is the first study showing that the association with all-cause and cardiovascular mortality is stronger with albuminuria than with dipstick proteinuria. This finding suggests that albuminuria is superior for predicting which subjects are at high risk for a poor prognosis at the health check.

One of the reasons for the difference in the association between albuminuria, dipstick proteinuria, and mortality might be that the urine examination results are adjusted for urine concentration in the diagnosis of albuminuria, but not of proteinuria by dipstick. Johnston et al. reported that the urine concentration greatly varies under routine clinical conditions [11], suggesting the results of evaluations of urine samples without adjustment for urine concentration might be less accurate. Therefore, it is strongly recommended to evaluate albuminuria and proteinuria only after adjusting for the urine concentration of creatinine [12]. Additionally, the dipstick reacts to nonalbumin proteins such as alpha1-, beta2-, and gamma-globulin and Bence-Jones protein. Low-grade proteinuria may not always indicate albuminuria. Furthermore, it is possible that the visual judgement of the grade of proteinuria, the pH of urine sample and the concomitant use of drugs such as ranitidine, acetazolamide and several types of antibiotics might influence the result of dipstick test. This speculation is partly supported by the finding that 11.9–25.2 % of the subjects in this study with proteinuria did not have albuminuria.

Of note, the prevalence of albuminuria and proteinuria varied greatly in this study (14.9 % in albuminuria, 8.4 % in ≥trace proteinuria, and 4.4 % in ≥[1+] proteinuria). This suggests that the number of subjects identified as being at high risk for ESKD, CVD, and death is affected by the method and cutoff point of the screening test used. Previously, we reported that the median urinary ACR was 43 mg/g for trace proteinuria and 81 mg/g for (+1) proteinuria [5], indicating that the threshold levels of ACR for albuminuria might be lower than those for trace and (1+) proteinuria. This could be one of the main reasons for the high prevalence of albuminuria compared with proteinuria in this population.

Although our results suggest that albuminuria can detect high-risk subjects, the cost of directly measuring the urine albumin concentration is high. In Japan, the cost of evaluating albuminuria and proteinuria by dipstick is US $10 and US $0.80 (1 US $ = 120 Japanese Yen), respectively. In addition, the results obtained by dipstick are available on the spot, but not for the direct measurement of albumin concentration in urine. Therefore, the dipstick test has an advantage in the cost-effectiveness and the convenience of the examination. At a health check, these points together with the diagnostic performance should be considered.

This study has several limitations. First, we evaluated the levels of albuminuria and proteinuria at baseline only once. Because these values often show a day-to-day variation, we might have underestimated the association between albuminuria/proteinuria and prognosis. Second, the cause of albuminuria and proteinuria is not clarified in this study. It has been reported that the risk of cardiovascular events is different depending on the origin of renal disease [13]. Third, we included various confounders such as age, gender, dietary and living habits, and comorbidities in our multivariate analyses. However, there is a possibility that other known and unknown factors may affect the association observed in this study.

In conclusion, the prevalence of albuminuria and its ability to predict 7-year all-cause and cardiovascular mortality were higher than that of proteinuria by dipstick, suggesting that albuminuria might be a superior predictor of prognosis in local inhabitants at a health check.
